# CCN2 reduction mediates protective effects of BMP7 treatment in obstructive nephropathy

**DOI:** 10.1007/s12079-016-0358-2

**Published:** 2016-10-20

**Authors:** Lucas L. Falke, Jan Willem Leeuwis, Karen M. Lyons, Christine L. Mummery, Tri Q. Nguyen, Roel Goldschmeding

**Affiliations:** 1 Department of Pathology, Kidney Group, University Medical Centre Utrecht, H04.312, Heidelberglaan 100, 3584 CX Utrecht, The Netherlands; 2 Department of Molecular, Cell and Developmental Biology, University of California, Los Angeles, CA USA; 3 Department of Anatomy and Embryology, Leiden University Medical Centre, Leiden, The Netherlands

**Keywords:** BMP7, TGFβ, CCN2, CKD, UUO, Kidney

## Abstract

Treatment with rhBMP7 exerts profound protective effects in a wide variety of experimental models of renal disease. However, little is known about how these protective effects are mediated, and which cells in the kidney are targeted by exogenous rhBMP7 treatment. To determine if rhBMP7 increases glomerular and tubulointerstitial canonical BMP signaling, we performed Unilateral Ureteral Obstruction (UUO, a widely used obstructive nephropathy model) in mice reporting transcriptional activity downstream of canonical BMP signaling by the expression of GFP under the BMP Responsive Element of the Id1 promoter (BRE:gfp mice). We also analysed the impact of rhBMP7 treatment on severity of the UUO phenotype, on TGFβ signaling, and on expression of CCN2 (CTGF). Despite profound protective effects with respect to morphological damage, macrophage infiltration, and fibrosis, no significant difference in GFP-expression was observed upon rhBMP7 administration. Also TGFβ signalling was similar in rhBMP7 and vehicle treated mice, but CCN2 expression in obstructed kidneys was significantly reduced by rhBMP7 treatment. Of note, in heterozygous CCN2 mice (CCN2+/−) treatment with rhBMP7 did not (further) reduce the severity of kidney damage in the UUO-model. These data suggest that protection against obstructive nephropathy by exogenous rhBMP7 treatment relies primarily on non-canonical BMP signaling, and may be mediated in large part by downregulation of CCN2 expression.

**Electronic supplementary material:**

The online version of this article (doi:10.1007/s12079-016-0358-2) contains supplementary material, which is available to authorized users.

## Introduction

Irrespective of underlying aetiology, chronic kidney disease (CKD) involves structural changes, and ultimately loss of function and fibrosis. Although, there is no effective treatment, several potential targets for intervention in CKD progression have been identified. Transforming Growth Factor beta (TGFβ) is generally regarded as the main culprit driving CKD progression (Meng et al. 2015). Numerous studies targeting TGFβ in various experimental diseases have yielded favourable results, but recent clinical trials have questioned efficacy of available interventions in human CKD (Akhurst and Hata 2012; Muñoz-Félix et al. 2015) (Clinicaltrial.gov numbers NCT00464321 and NCT01113801).

The administration of recombinant human BMP7 (rhBMP7; Bone Morphogenetic Protein 7) has been proposed as an attractive alternative intervention to stop progression of CKD. Several landmark papers have shown efficacy of BMP7 treatment in a wide range of experimental models of renal disease including diabetic nephropathy, obstructive uropathy, nephron loss and ischemic injury (Vukicevic et al. 1998; Hruska et al. 2000; Morrissey et al. 2002; Wang et al. 2003; Zeisberg et al. 2003; Dube et al. 2004; Sugimoto et al. 2007), and a BMP-mimetic (THR-185) is under study in a phase II clinical trial (Clinicaltrial.gov number NCT01830920). BMP7 treatment is considered to attenuate experimental CKD at least in part by antagonizing TGFβ (Wang and Hirschberg 2003; Zeisberg et al. 2003). BMP7 is required for kidney development and remains highly expressed during adult life (Dudley et al. 1995). Although also several other BMPs are expressed in the kidney throughout development and adulthood, including BMP4 and BMP6, the potential therapeutic effects of BMP7 are considered most potent (Zeisberg et al. 2003; Dendooven et al. 2011).

Despite all evidence supporting BMP7 efficacy in CKD, the identity and localization of cells responding to exogenous BMP7 treatment remain to be identified. Previous studies in BMP canonical signaling reporter mice (BRE:gfp mice) identified glomerular and collecting duct cells to have high endogenous BMP signalling activity (Leeuwis et al. 2011). Signaling activity in the glomeruli and medulla dropped upon Unilateral Ureteral Obstruction (UUO), but it increased in the proximal tubular compartment. However, if and to what extent exogenous BMP7 therapeutic efficacy might involve restoration of canonical signaling activity in these particular cells or other nephron segments or cell types is still unclear.

CCN2, also known as Connective Tissue Growth Factor (CTGF) is yet another factor involved in CKD progression (Falke et al. 2014). CCN2 contributes to fibrosis by modulating signaling activity in BMP7, TGFβ, and other signaling pathways (Abreu et al. 2002; Nguyen et al. 2008)*.* CCN2 expression is increased in essentially all progressive kidney diseases, and CCN2 inhibition decreases loss of function and fibrosis (Ito et al. 1998; Falke et al. 2014; Ren et al. 2015). CCN2 can bind to BMP7 thereby inhibiting canonical SMAD1/5/8 signalling, and as such might also be an important determinant of the efficacy of BMP7 treatment (Nguyen et al. 2008).

In this study, we set out to shed more light on the mode of action of exogenous BMP7 therapy by analysing distribution of transcriptional activity downstream of canonical BMP signaling, and the associated complex interplay between BMP7, TGFβ, and CCN2 in rhBMP7 treated BRE-GFP reporter mice subjected to UUO.

## Materials and methods

### Animals

Generation of bone morphogenetic protein responsive element (BRE); Green Fluorescent Protein (GFP) reporter mice has been described in detail elsewhere (Monteiro et al. 2008). Briefly, these mice express GFP under control of the BRE in the Id1 gene promoter, thus reporting transcriptional activity downstream canonical BMP signaling. Male BRE:gfp mice on a C57Bl6/J background were used for this study. CCN2 hemizygous KO mice were used to estimate the relative contribution of CCN2 reduction to the therapeutic effect of exogenous BMP7. CCN2 (hemizygous) KO mice have been described previously (Ivkovic et al. 2003). Mice were kept on a 12-h light/day cycle with food and water ad libitum. All work was carried out with approval of the Experimental Animal Ethics Committee of the University of Utrecht.

### Unilateral ureteral obstruction/BMP7 administration

Mice were subjected to Unilateral Ureteral Obstruction under general isoflurane anaesthesia. The left flank was incised and the ureter was exposed and tied off using silk sutures. Directly after ligation, a depot of 300 μg/kg rhBMP7 (dissolved in PBS; kindly provided by Stryker, Kalamazoo, MI) per mouse was left intraperitoneally (i.p.). The wound was closed using 5–0 vicryl sutures (Ethicon, Sommerville, NJ). Mice received an additional 300 μg/kg rhBMP7 i.p. on day 2, 4 and 6 (*n* = 9). Vehicle (PBS) injected mice were used as control (*n* = 6). 3 mice were used for CCN2 hemizygous groups. At day 7 mice were killed by ketamin, xylazine and acepromazine overdose. Kidney tissue was fixed in fresh 4 % paraformaldehyde solution and embedded in paraffin, or snap frozen and stored at −80 °C until further processing.

### (Immuno)histochemistry

Sections (3 μm) were cut from paraffin blocks, deparaffinised and rehydrated. For assessment of morphological changes, sections were stained with Periodic Acid Schiff using standard methods. 10 random cortical fields of PAS stained sections were scored on a five-point scale for tubular atrophy or dilatation (0 = 0–10 %, 1 = 10–25 %, 2 = 25–50 %, 3 = 50–75 %, 4 = 75–100 %). Masson Tri Chrome (MTC) and Sirius Red staining was performed using standardized protocols at the clinical diagnostics laboratory of our department.

Direct fluorescence of 20 glomeruli per kidney was assessed in images acquired by confocal laser scanning microscopy (CLSM), followed by ImageJ analysis of signal intensity. Likewise, direct tubuloinsterstitial GFP signal was assessed in CLSM images by analyzing area positivity and single channel pixel intensity of 10 cortical fields from which glomeruli were deselected.

For immunohistochemistry, slides were boiled in EDTA or citrate buffer for antigen retrieval where appropriate, and incubated with the following antibodies: anti-GFP (Ab290 , rabbit polyclonal, Citrate, 1:1000, Abcam, Cambridge, UK), HRP-linked LTA (Citrate, 1:32, Sigma-Aldrich, St. Louis, MO), anti-CCN2 (L20 , goat polyclonal, Citrate, 1:200, Santa-Cruz Biotechnology, Santa Cruz, CA), anti-F4/80 (Fresh Frozen tissue, rat monoclonal, 1:3000, Serotec/Biorad antibodies, Oxford, UK) or anti-αSMA (Ab5694, rabbit polyclonal, EDTA, 1:200, Abcam). Ready to use Horse Radish Peroxidase (HRP) linked BrightVision (ImmunoLogic, Duiven, NL) species specific secondary antibody solution, followed by NovaRed (Vector laboratories, Burlingham, CA) or 3,3′-diaminobenzidine (Sigma Aldrich, St. Louis, MO) were applied for chromogen color development to visualize bound primary antibody.

The number of proximal tubule cross sections per cortical surface area was counted in 10 random fields per kidney (200× magnified). Using Photoshop (Adobe, San Jose, CA), a colour selection of positively stained area was made after which pictures were dichotomized in IHC positive and staining negative pixels. Using this method, the percentage positive cross sectional surface area for GFP, F4/80, and αSMA respectively, was determined in 10 cortical fields photographed at 200× magnification by ImageJ analysis (NIH).

For glomerular GFP IHC area positivity, pictures of 20 glomeruli per kidney were converted into binary images. Supplemental Fig. 1 A shows one example of a binary image of the CLK from the vehicle group illustrating what is regarded as total and positive area for calculation of positive area percentage. The red dotted lines define the circumference of two glomeruli. The enclosed total glomerular cross-sectional surface is used for calculation of glomerular positive area percentage. The total glomerular cross-sectional surface area is excluded for calculation of tubulointerstitial positive area percentage.

### RT-qPCR

Full Kidney cortex mRNA was isolated using Trizol and 3000 ng of mRNA was reversely transcribed into cDNA. RT-qPCR was performed on a LightCycler480 (Roche, Basel, Switzerland), using commercially available TaqMan primer assays (Thermo Fisher/Life technologies, Waltham, MA): (Yhwaz; Mm03950126_s1, Ctgf, Mm00515790_g1; Tgfβ1, Mm01178820_m1; Col1α2, Mm00483888_m1; Pai1, Mm00435860_m1). Sybr green GFP primers and probe were designed with Primer Express (Applied Biosystems, Foster City, CA) and purchased from Eurogentec (Maastricht, The Netherlands) (primers) and Applied Biosystems (probe). *Yhwaz* expression was used as internal reference. Relative expression values were calculated using the ΔΔCT method.

### Statistics

All statistical analyses were performed using Graphpad Prism (GraphPad, LaJolla, CA). ANOVA with Tukey post-hoc correction for multiple testing or Student T-test were used where appropriate. A *p*-value below 0.05 was considered statistically significant.

## Results

### Exogenous BMP7 does not increase BRE:gfp reporter signal for transcriptional activity downstream of canonical BMP7 signaling

**Fig. 1:**
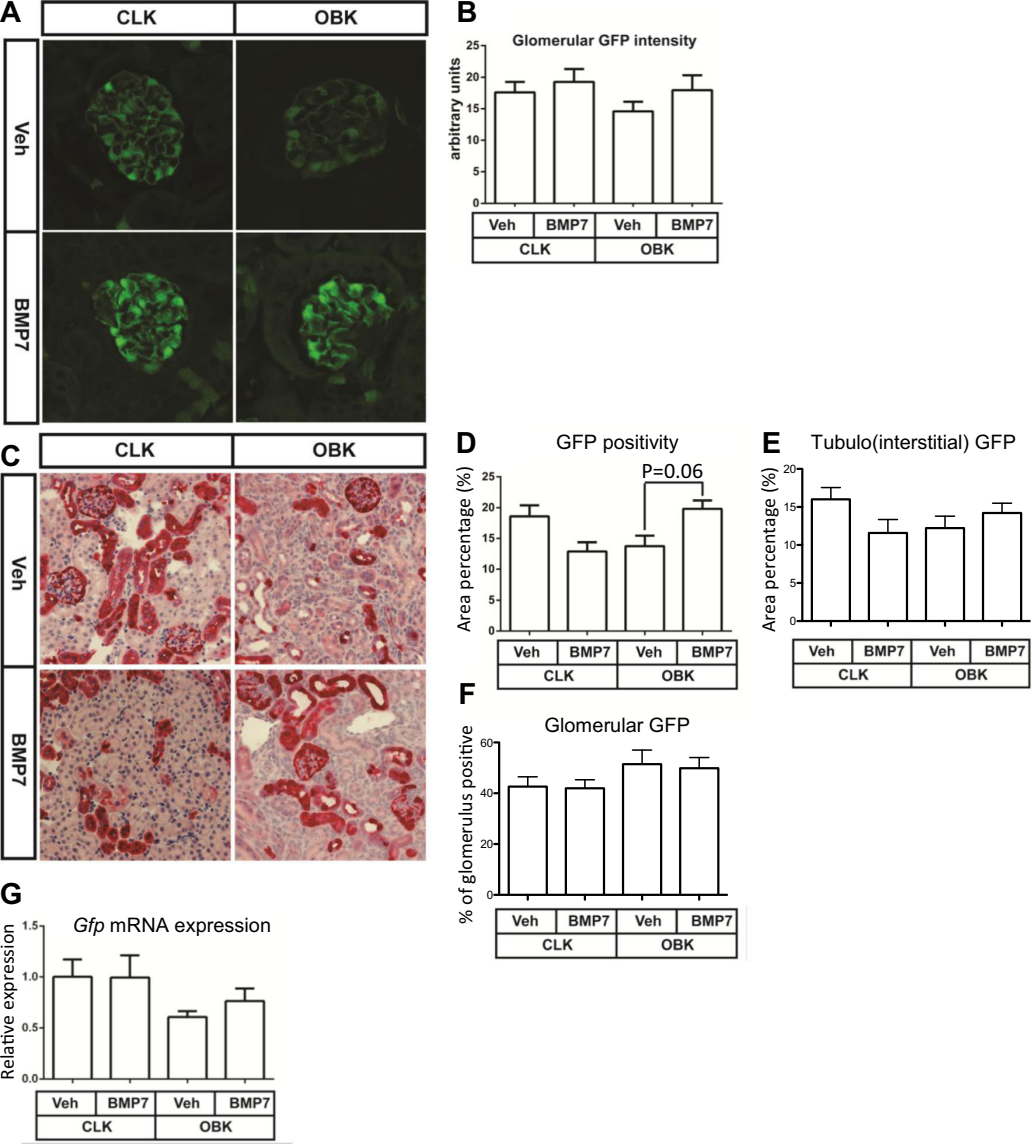
rhBMP7 treated BRE:Gfp OBKs do not show increased GFP expression. **a** Representative CLSM micrographs of direct glomerular GFP expression (pseudo colorized). **b** Quantification of GFP intensity. **C.** Representative images of GFP immunohistochemistry in CLKs and OBKs 7 days after UUO of both vehicle and BMP7 groups (200× magnified). **d-f.** Quantification of total area (B), tubules and interstitium (C) or percentage of the glomeruli (D) positive for GFP. **G.** Cortical *Gfp* mRNA expression. Error bars represent SEM

Body weight, kidneys weights and kidney weight/body weight ratios of rhBMP7 treated BRE:gfp mice were not significantly different from those in vehicle treated mice (See Table 1). BRE:gfp mice express GFP under a SMAD binding element in the Id1 promotor region and thereby report transcriptional activity downstream of canonical BMP signaling (Korchynskyi and Dijke ten 2002; Monteiro et al. 2008). Glomerular GFP intensity tended to be slightly lower in obstructed kidneys of vehicle treated mice (*P* = 0.3; Fig. 1a, b). Also, in the tubulointerstitial compartment of the kidney cortex there was a trend towards reduction of GFP-positive surface area, and GFP signal intensity was slightly reduced upon obstruction (Suppl. Figure 1a–c). Remarkably, rhBMP7 treatment induced only very limited, non-significant increase of glomerular GFP fluorescence and altered neither total GFP positive surface area nor fluorescent signal intensity in GFP positive tubulointerstitial areas (Fig. 1a, b, Suppl. Figure 1a–c respectively).

**Table 1:** Body and kidney weights of both treatment groups. Average (+/− standard deviation) is shown

	Vehicle (n = 6)	rhBMP7 (n = 9)	*p*-value
BW start (g)	**25.92** (+/−4.41)	**25.88** (+/− 4.32)	0.99
BW end (g)	**25.87** (+/− 3.82)	**24.84** (+/− 3.52)	0.61
BW end/BW start	**1** (+/− 0.05)	**0.96** (+/−0.04)	0.15
CLKW (mg)	**211.6** (+/−48.2)	**211** (+/− 47.69)	0.98
OBKW (mg)	**194.67** (+/− 37.33)	**199.88** (+/− 25.98)	0.76
CLKW (mg/g BWs)	**8.12** (+/− 0.8)	**8.1** (+/− 0.67)	0.96
OBKW (mg/g BWs)	**7.52** (+/− 0.9)	**7.81** (+/−0.96)	0.58
OBKW/CLKW (mg/mg)	**0.91** (+/− 0.08)	**0.97** (+/− 0.14)	0.41

Body and kidney weights of both treatment groups. Average (+/− standard deviation) is shown.

*BW* body weight, *CLKW* contralateral kidney weight, *OBKW* obstructed kidney weight

Comparison between GFP signal detected by direct fluorescence and immunohistochemically (IHC) revealed that IHC detection is more sensitive (Suppl. Figure 2). Using morphometric analysis, percentage positive area of total cortical sections, tubulointerstitial (TI) compartment and glomeruli was analysed in sections where GFP was detected by ImageJ, thus allowing assessment whether canonical BMP signaling increases in cortical areas where normally signalling is absent or below detection limit (Suppl. Figure 3). Average total and TI GFP positive area tended to be lower in OBKs than in CLKs, but the observed difference was not significant (Fig. 1d, e). Remarkably, also the apparently small increase of total cortical GFP positive area in rhBMP7 treated OBKs was not statistically significant (Fig. 1d; *p* = 0.06), and totally lost when tubules and glomeruli were analysed separately (Fig. 1e, f). Analysis of cortical *Gfp* mRNA expression revealed a significant decrease upon ureteral obstruction, but also here no effect of BMP7 treatment was observed (Fig. 1g).

### BMP7 protects against kidney damage and reduces macrophage infiltration 7 days post-UUO

**Fig. 2:**
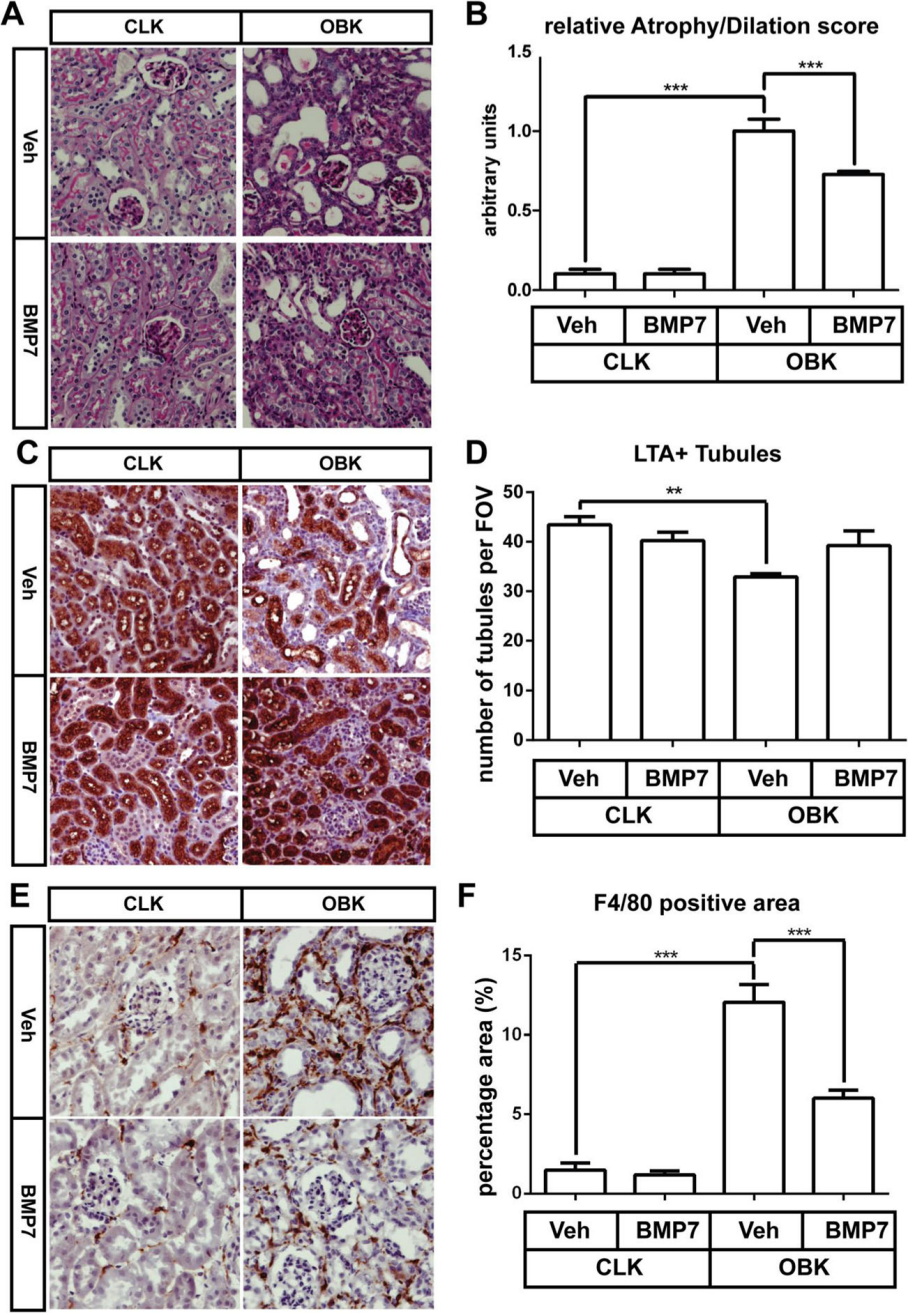
rhBMP7 treatment conserves renal morphology and limits macrophage accumulation 7 days after obstructive nephropathy. **a** Representative images of PAS stained cortical sections in CLKs and OBKs 7 days after UUO of both vehicle and BMP7 treated groups (200× magnified). **b** Composite of atrophy and dilatation score quantified on PAS stained slides **c** Representative images of LTA stained cortical sections in CLKs and OBKs of both vehicle and BMP7 treated groups (200× magnified). **d** Quantification of the average number of LTA+ proximal tubules per HPF in CLKs and OBKs of vehicle and rhBMP7 treated kidneys. **e** Representative images of F4/80 stained cortical sections in CLKs and OBKs of both vehicle and BMP7 treated groups (200× magnified). **f** Positive area quantification of F4/80 positive macrophages in CLKs and OBKs of vehicle and rhBMP7 treated kidneys. *p < 0.05, ***p < 0.005. Error bar represents SEM. Vehicle *n* = 6, rhBMP7 *n* = 9

In PAS stained kidney sections the increase of tubular atrophy and dilatation in OBKs was attenuated in BMP7-treated mice (*p* < 0.005; Fig. 2a, b). *Lotus tetragonolobus* Agglutinin (LTA) staining revealed that the number of LTA+ proximal tubules was decreased in OBKs compared to CLKs, but less so in BMP7 treated mice (Fig. 2c, d; *p* < 0.01) Additionally, the OBK/CLK ratio was significantly higher in rhBMP7 treated animals (*p* < 0.05).

Macrophage infiltration in OBKs, as assessed by F4/80 IHC, was markedly reduced in the BMP7 treated group (p < 0.005; Fig. 2e, f).

### Fibrogenesis is reduced in BMP7 treated OBKs

**Fig. 3:**
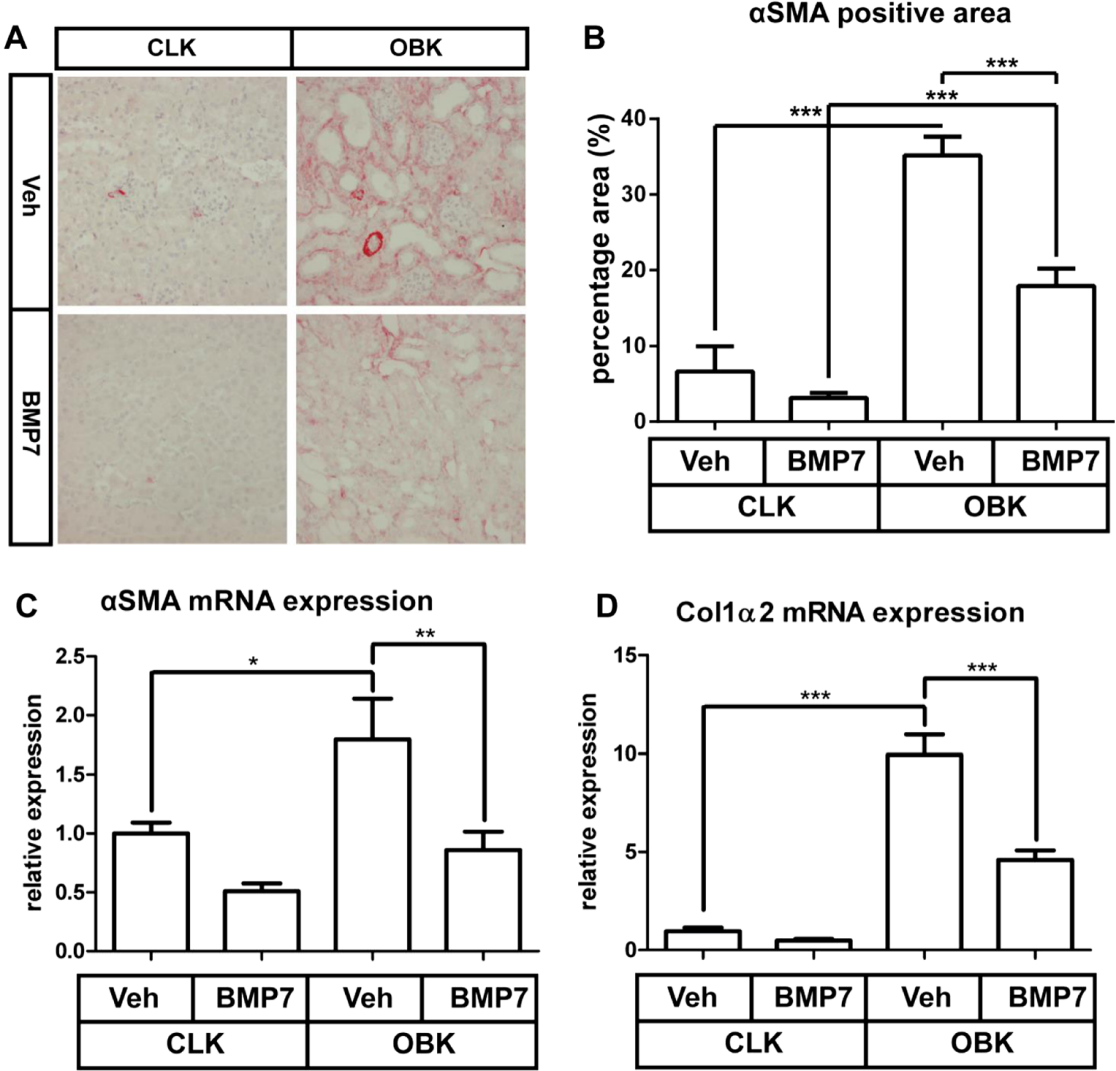
rhBMP7 treatment reduces myofibroblast accumulation and associated de novo collagen production. **a** Representative images of αSMA in CLKs and OBKs 7 days after UUO of both vehicle and BMP7 treated groups (200× magnified). **b** Positive area quantification of αSMA positive myofibroblasts in CLKs and OBKs of vehicle and rhBMP7. **c** and **d** *αSma* mRNA (C) and Col1α2 (D) expression levels in kidney cortex of CLKs and OBKs 7 days after UUO of vehicle and rhBMP7 treated animals. *p < 0.05, **p < 0.01, ***p < 0.005. Error bars represent SEM

After 7 days of obstruction, the OBKs of vehicle treated mice showed only a little increase in fibrosis (Suppl. Figure 4). Accumulation of myofibroblasts in OBKS, as assessed by αSMA positive surface area, was decreased by BMP7 treatment (p < 0.005; Fig. 3a, b). Furthermore, the increase of mRNA for *α-sma* and *Col1α2* was reduced by BMP7 treatment (p < 0.01 and p < 0.005 resp.; Fig. 3c, d).

### BMP7 treatment reduced CCN2 expression but did not alter Tgfβ1expression and transcriptional activity

**Fig. 4:**
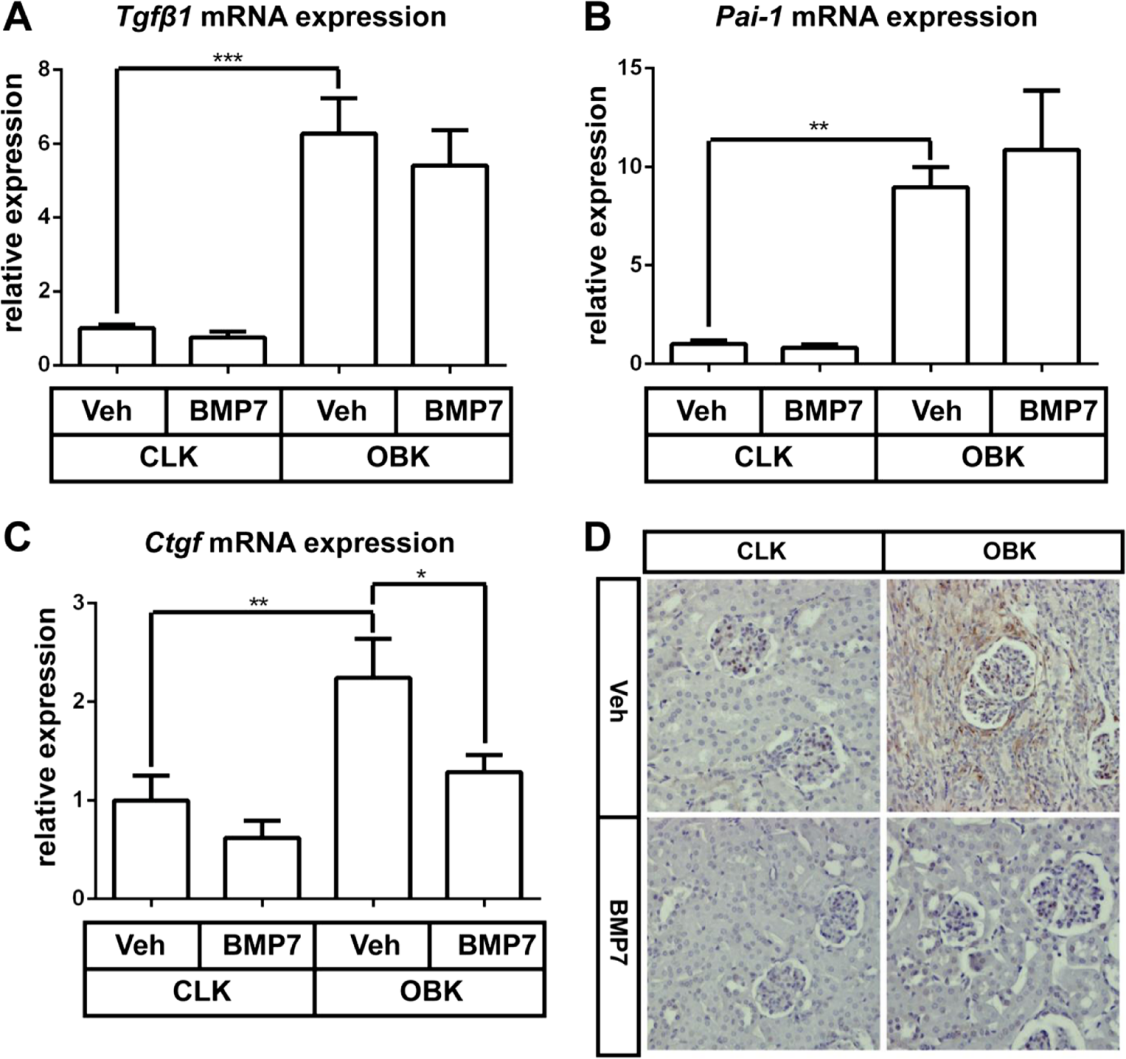
rhBMP7 treatment reduces CCN2 expression levels without altering canonical TGFβ signaling. **a-c** Cortical mRNA expression levels of Tgfβ1 (A), Pai-1 (B) and CCN2 (C) in CLKs and OBKs 7 days after UUO of vehicle and rhBMP7 treated CCN2+/− mice. **d** Representative images of CCN2 immunohistochemistry in CLKs and OBKs of both vehicle and rhBMP7 treated groups (200× magnified). *p < 0.05, **p < 0.01, ***p < 0.005. Error bars represent SEM

Both *Tgfβ1* and its canonical transcriptional target *Pai-1*/*SerpinE1* were upregulated in OBKs (p < 0.005 and p < 0.01 resp.; Fig. 4a, b), which was not affected by BMP7 treatment. However, BMP7 blocked the increase of CCN2 expression in OBKs (p < 0.05; Fig. 4c, d).

### In CCN2 hemizygous KO mice, BMP7 treatment did not further reduce damage, macrophage infiltration, and fibrosis of obstructed kidneys

**Fig. 5:**
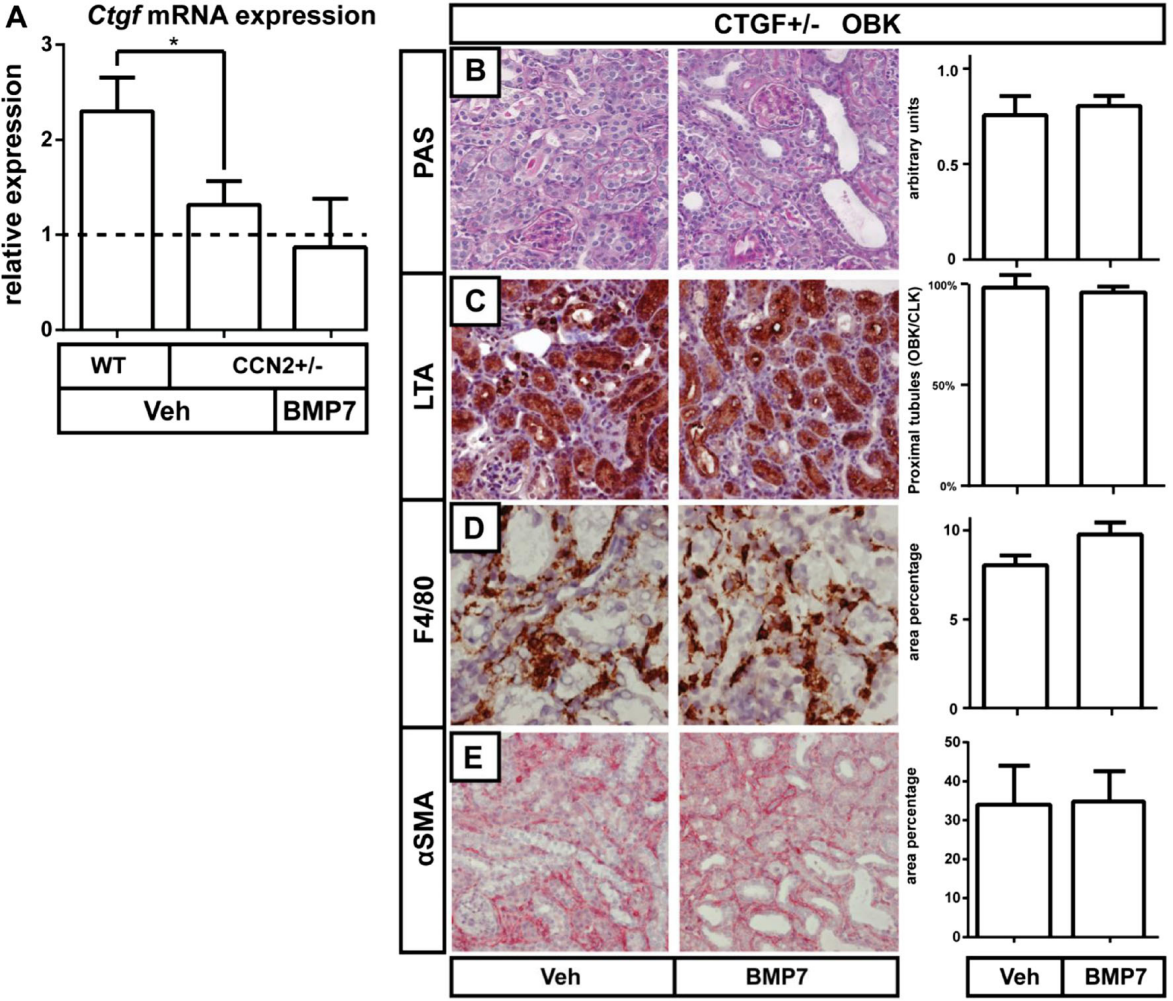
Treatment with rhBMP7 offers no additional renoprotection in CCN2+/− heterozygous KO mice. **a** Average cortical mRNA expression levels of CCN2 in CLKs and OBKs 7 days after UUO of vehicle and rhBMP7 treated mice. **b-e.** Representative micrographs (200× magnified) and corresponding quantification of morphological composite damage score (B), LTA positive proximal tubules (C), F4/80 positive macrophages (D) and αSMA positive myofibroblasts (E), in OBKs 7 days after UUO of vehicle and rhBMP7 treated CCN2+/− mice. *p < 0.05. Error bars represent SEM

In good agreement with previous reports, we observed less severe kidney damage, macrophage infiltration, and fibrosis in 7 day OBKs of CCN2 hemizygous KO, than in those of wild type mice. (Suppl. Figure 5a–e). In order to investigate BMP7 induced reno-protection beyond *CCN2* reduction, we also treated CCN2+/− UUO mice with rhBMP7. The approximately 50 % reduction of *CCN2* expression in CTGF+/− mice was not further reduced by rhBMP7 treatment (Fig. 5a). In association with this finding, administration of BMP7 to CCN2 hemizygous KO mice also failed to further attenuate renal damage, macrophage infiltration or myofibroblast accumulation (Fig. 5b–d), which emphasizes the importance of CCN2 reduction in mediating the protective effect of BMP7 treatment in this model of obstructive nephropathy.

## Discussion

The present study confirms that rhBMP7 treatment reduces the severity of kidney damage, macrophage infiltration, and myofibroblast accumulation in a mouse model of obstructive nephropathy (UUO). Seven days after UUO, little to no manifest fibrosis was present (Suppl. Figure 3), which might reflect the relative resistance of C57Bl6 mice to renal fibrosis in general (Walkin et al. 2013). However, analysis of Col1α2 transcription revealed that de-novo collagen type 1 production was reduced in rhBMP7 treated mice. Remarkably, transcriptional activity downstream of canonical BMP signaling, and also TGFβ expression and transcriptional activity, appeared not to be altered by rhBMP7 treatment, while the expression of CCN2 was significantly reduced in the obstructed kidneys (OBK) of BMP7-treated mice.

In line with previous observations in the BRE-GFP reporter mouse also used here, the GFP signal was slightly reduced in distal tubuli of obstructed kidneys (Fig. 1), but we detected no increase of GFP signal upon rhBMP7-treatment (Manson et al. 2015). Moreover, direct fluorescence of glomeruli and tubulointerstitium showed that established canonical BMP signalling was not increased in intensity nor in terms of positive surface area (Fig. 1a, b, Suppl. Figure 2a–d respectively). Also with more sensitive immunohistochemical detection of GFP protein detection, there was no increase of GFP positive area percentage, indicating that BMP7 did not induce de novo canonical BMP transcriptional activity in previously negative cells (Fig. 1c–f). Unfortunately given its non-stoichiometric nature, IHC signal intensity analysis was not feasible. However, the lack of increase of transcriptional activity downstream of canonical BMP signaling was further underlined by equal GFP mRNA expression levels in renal cortex of rhBMP7 treated and untreated mice (Fig. 1g). It thus appears that the protective effects of rhBMP7 treatment in the 7 days UUO model does not require or involve an increase of canonical BMP transcriptional activity. This suggests that the beneficial effects of BMP7 treatment mainly involve non-canonical signaling, rather than transcriptional activity downstream of canonical BMP signaling. However, since no data are available on pharmacokinetics of i.p. injected rhBMP7, we cannot fully exclude that rapid elimination or degradation of de novo synthesized GFP protein within the 24 h window between the last BMP7 dose and sacrifice might have “quenched” the reporter signal.

Previously, we have shown that BMP7 treatment of renal interstitial fibroblasts reduces CCN2 and PAI-1 expression (Nguyen et al. 2006), and Wang et al. demonstrated that BMP7 treatment of mesangial cells reduces CCN2, Fibronectin and Collagen type 4 expression (Wang and Hirschberg 2003). BMP7 also exerts protective effects on TGF- β stimulated podocytes (Mitu et al. 2007). As for proximal tubular epithelial cells, Zeisberg et al. showed that BMP7 can inhibit and even reverse TGFβ driven epithelial to mesenchymal transdifferentiation, although this could not be confirmed by Dudas et al. (Zeisberg et al. 2003; Dudas et al. 2009). In aggregate, it thus appears that rhBMP7 can reduce (TGFβ-induced) CCN2 expression and associated fibrotic changes in most cell types of the kidney. However, most evidence for this derives from in vitro cultures only, and the relative contribution of canonical as compared to non-canonical signaling in this has not yet been clearly delineated. The renoprotective effects of rhBMP7 were associated with approximately 50 % reduction of CCN2 expression, whereas expression of *Tgfβ1* and its prototypic canonical transcriptional target *Pai-1* remained unaltered (Fig. 4). Efficacy of BMP7 therapy without altering TGFβ signaling has been reported previously in a model of diabetic nephropathy (Wang et al. 2006). Of note, hemizygous CCN2 deletion (with 50 % reduced CCN2 expression), appeared equally effective as rhBMP7 treatment, with similar reduction of morphological damage, macrophage infiltration, and collagen and α-SMA expression (Suppl. Figure 5). This is consistent with previous observations that an approximately 50 % reduction of CCN2 expression by genetic deletion or siRNA was sufficient to significantly attenuate models of diabetic nephropathy and obstructive nephropathy (Yokoi et al. 2004; Guha et al. 2007; Nguyen et al. 2008). With respect to possible concerns regarding potential a confounding effect of CCN2-suppression prior- and unrelated to the induction of obstructive nephropathy, the reduced CCN2 expression in conventional CCN2+/− mice, did not lead to any renal abnormalities during development or adult life, and neither did a 90 % CCN2 reduction sustained for several weeks (Ivkovic et al. 2003; Falke et al. 2012; Falke et al. 2014).

Interestingly, administration of rhBMP7 to heterozygous CCN2 mice tended to even further reduce CCN2 expression, but this was not associated with a further decrease in damage and fibrosis. It thus appears that the observed therapeutic effects might relate to a threshold effect of CCN2 reduction rather than on a continuous dose-response relation (Fig. 5).

In summary, in the mouse UUO model of obstructive nephropathy we observed efficacy of rhBMP therapy in the absence of clear evidence for modulation of transcriptional activity downstream of canonical BMP signaling. As a consequence, the BRE-GFP reporter mice failed to reveal the identity of specific phenotypes and localization of cells responding to exogenous BMP7 therapy. Furthermore, efficacy of BMP7 treatment appeared not to require reduction of TGFβ expression or transcriptional activity, but was associated with a reduction of CCN2 expression. Hemizygous deletion of CCN2 was able to reduce UUO severity and BMP7 had no additional effect. Together, these data suggest that protection against obstructive nephropathy by exogenous BMP7 treatment relates primarily to non-canonical BMP signalling. Possibly, BMP7 effects are mediated by downregulation of CCN2 expression.

## Electronic supplementary material

Supplemental Figure 1 (DOCX 10.4 mb)

Supplemental Figure 2 (DOCX 506 kb)

Supplemental Figure 3 (DOCX 217 kb)

Supplemental Figure 4 (DOCX 1074 kb)

Supplemental Figure 5 (DOCX 27 kb)

## Abbreviations

BMP7: Bone Morphogenetic Protein 7
CCN2: Cyr61-CTGF-Nov family protein 2
CTGF: Connective Tissue Growth Factor
CKD: Chronic Kidney Disease
UUO: Unilateral Ureteral Obstruction
BW: Body Weight
OBK: Obstructed Kidney
CLK: Contralateral Kidney
GFP: Green Fluorescent Protein
TGFβ: Transforming Growth Factor β
LTA: *Lotus tetragonolobus* Agglutinin
