# Supplementary material for: CCN2 reduction mediates protective effects of BMP7 treatment in obstructive nephropathy
Source: J Cell Commun Signal. 2016 Oct 20;11(1):39–48. doi: 10.1007/s12079-016-0358-2 (PMC5362571; doi:10.1007/s12079-016-0358-2)
Supplement: Supplementary file 1 — (DOCX 10.4 mb) [file 12079_2016_358_MOESM1_ESM.docx]

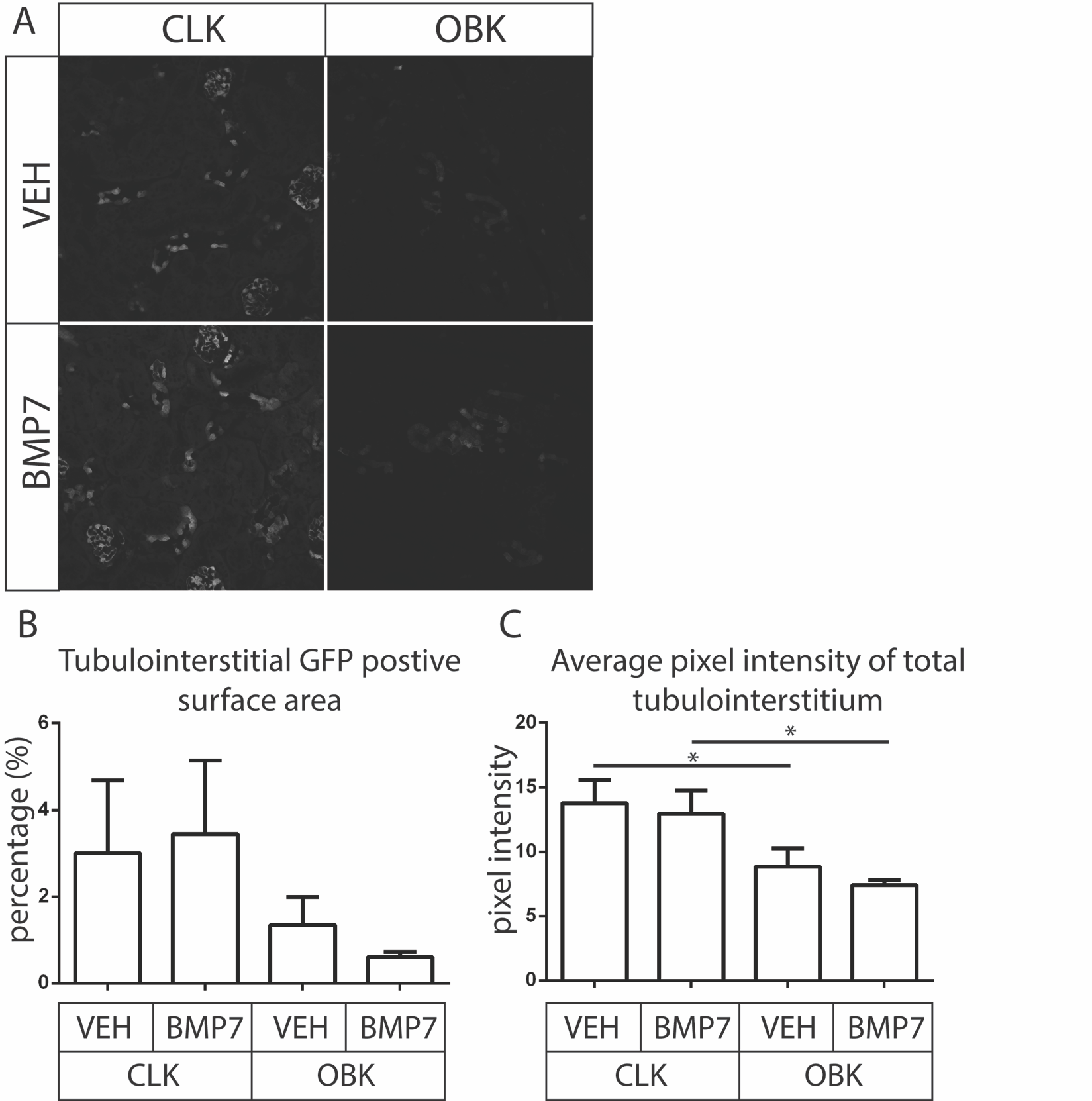


**Supplemental Figure 1:** Analysis of GFP expression area and intensity using confocal laser scanning microscope images. **A**) Representative CLSM images of direct single channel GFP signal. Quantification of **B**) total tubulointerstitial area positive for GFP (%) and **C**) average pixel intensity of total tubuloinsterstitium in both CLKs and OBKs of both treatment groups. * indicates P<0.05, Error bars represent SEM.
