# Supplementary material for: CCN2 reduction mediates protective effects of BMP7 treatment in obstructive nephropathy
Source: J Cell Commun Signal. 2016 Oct 20;11(1):39–48. doi: 10.1007/s12079-016-0358-2 (PMC5362571; doi:10.1007/s12079-016-0358-2)
Supplement: Supplementary file 2 — (DOCX 506 kb) [file 12079_2016_358_MOESM2_ESM.docx]

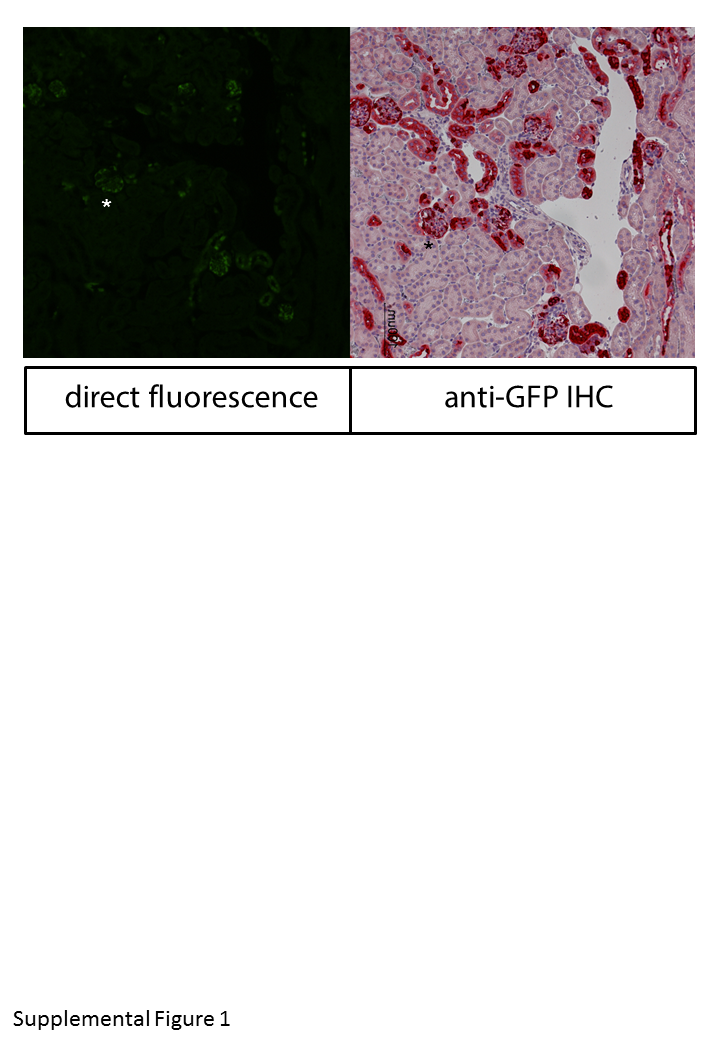


**Supplemental Figure 2:** Comparison between direct GFP fluorescence and immunohistochemically detected GFP in BRE;*gfp* kidney cortex. Asterisk indicates the same glomerulus in consecutive tissue sections. 100x magnified.
