# Supplementary material for: CCN2 reduction mediates protective effects of BMP7 treatment in obstructive nephropathy
Source: J Cell Commun Signal. 2016 Oct 20;11(1):39–48. doi: 10.1007/s12079-016-0358-2 (PMC5362571; doi:10.1007/s12079-016-0358-2)
Supplement: Supplementary file 3 — (DOCX 217 kb) [file 12079_2016_358_MOESM3_ESM.docx]

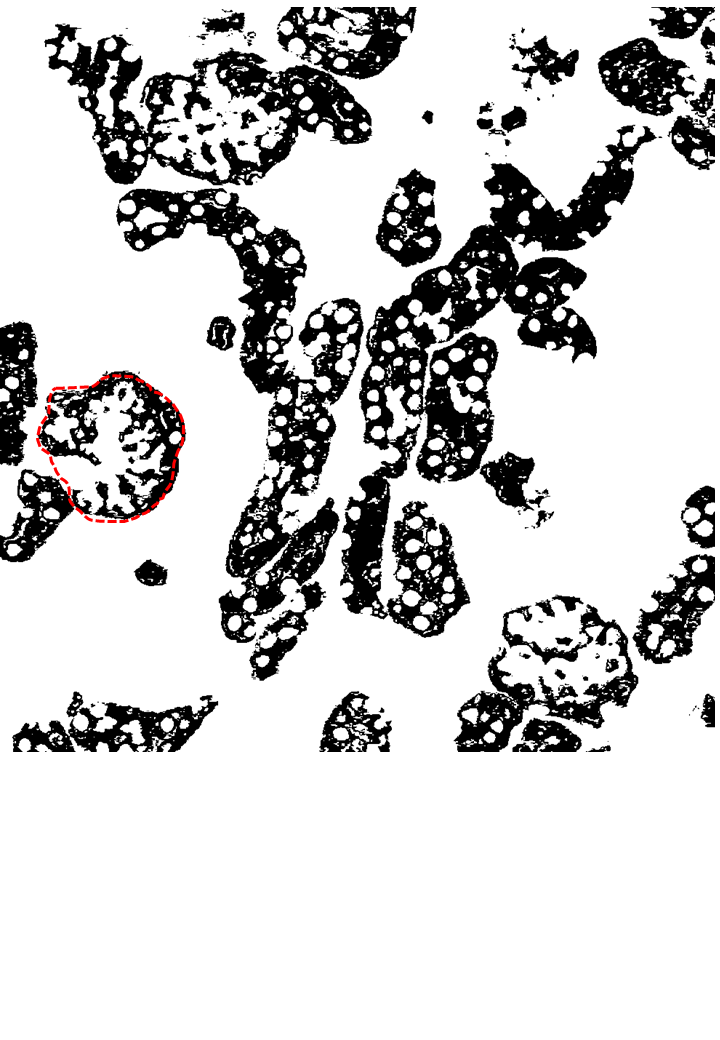


**Supplemental Figure 3**: Image resulting from Photoshop color selection and ImageJ conversion to binary image. Red dotted line illustrates methodology of tracing the circumference of a glomerulus for glomerular GFP positive surface area assessment.
