# Supplementary material for: CCN2 reduction mediates protective effects of BMP7 treatment in obstructive nephropathy
Source: J Cell Commun Signal. 2016 Oct 20;11(1):39–48. doi: 10.1007/s12079-016-0358-2 (PMC5362571; doi:10.1007/s12079-016-0358-2)
Supplement: Supplementary file 4 — (DOCX 1074 kb) [file 12079_2016_358_MOESM4_ESM.docx]

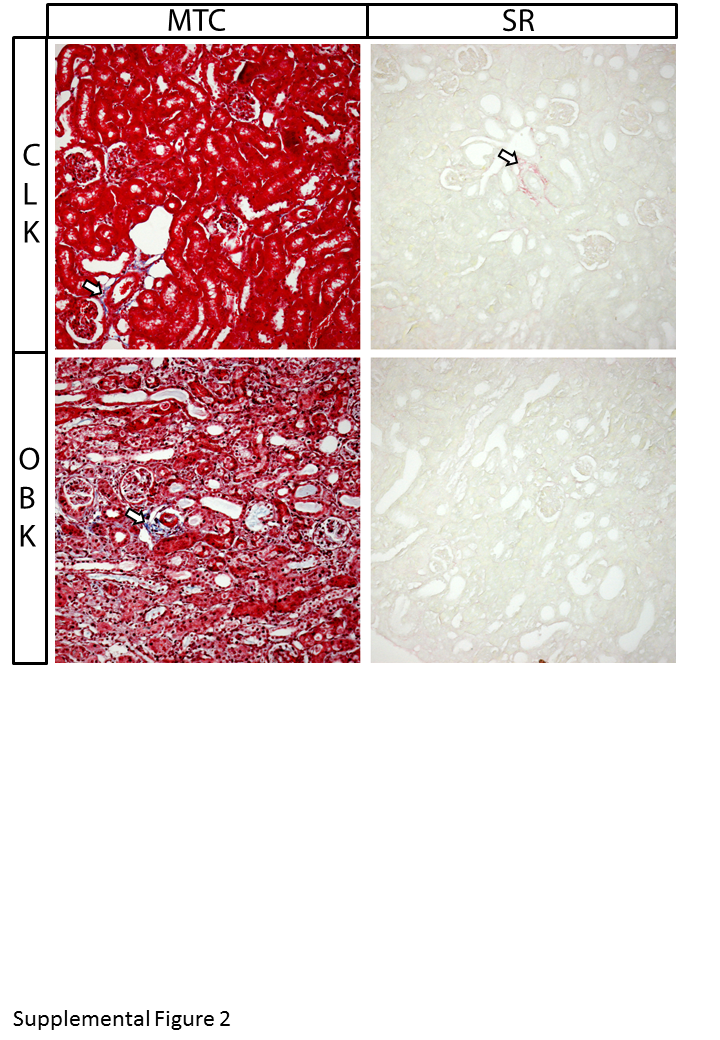


**Supplemental Figure 4**: Masson Trichrome (MTC) and Sirius Red (SR) histochemical detection of extracellular matrix deposition in CLKs and OBKs. Representative micrographs shown. Arrows indicate intracortical arteries with surrounding collagen deposition. 100x magnified.
