# Supplementary material for: CCN2 reduction mediates protective effects of BMP7 treatment in obstructive nephropathy
Source: J Cell Commun Signal. 2016 Oct 20;11(1):39–48. doi: 10.1007/s12079-016-0358-2 (PMC5362571; doi:10.1007/s12079-016-0358-2)
Supplement: Supplementary file 5 — (DOCX 27 kb) [file 12079_2016_358_MOESM5_ESM.docx]

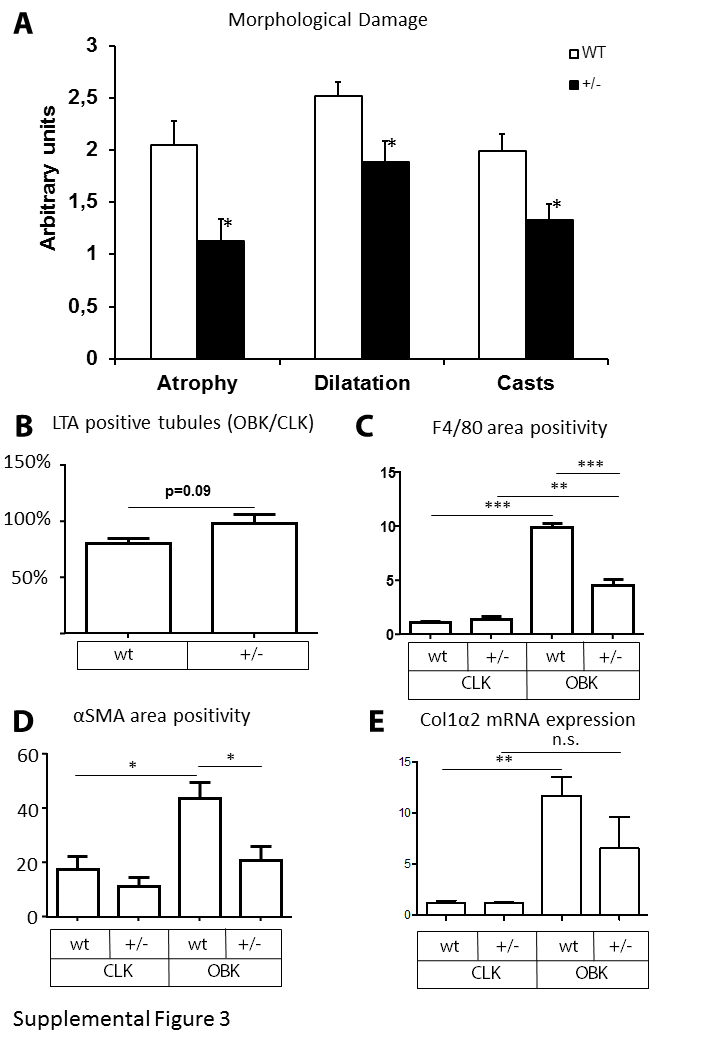


**Supplemental Figure 5**: Comparison of renal outcome between wild type and CTGF heterozygous KO (+/-) mice 7 days after UUO. Quantification of **A**) morphological damage, **B**) reduction in LTA positive proximal tubules, **C**) F4/80 positive macrophages **D**) αSMA positive myofibroblasts and **E**) Col1α2 mRNA expression. *p<0.05, **p<0.01, ***p<0.005. Error bar represents SEM.
